# Envelope Recombination: A Major Driver in Shaping Retroviral Diversification and Evolution within the Host Genome

**DOI:** 10.3390/v15091856

**Published:** 2023-08-31

**Authors:** Saili Chabukswar, Nicole Grandi, Liang-Tzung Lin, Enzo Tramontano

**Affiliations:** 1 Laboratory of Molecular Virology, Department of Life and Environmental Sciences, University of Cagliari, 09042 Cagliari, Italy; sailis.chabukswar@unica.it (S.C.); nicole.grandi@unica.it (N.G.); 2 International Ph.D. Program in Medicine, College of Medicine, Taipei Medical University, Taipei 110, Taiwan; 3 Department of Microbiology and Immunology, School of Medicine, College of Medicine, Taipei Medical University, Taipei 110, Taiwan; ltlin@tmu.edu.tw; 4 Graduate Institute of Medical Sciences, College of Medicine, Taipei Medical University, Taipei 110, Taiwan

**Keywords:** endogenous retroviruses, envelope gene, recombination

## Abstract

Endogenous retroviruses (ERVs) are integrated into host DNA as the result of ancient germ line infections, primarily by extinct exogenous retroviruses. Thus, vertebrates’ genomes contain thousands of ERV copies, providing a “fossil” record for ancestral retroviral diversity and its evolution within the host genome. Like other retroviruses, the ERV proviral sequence consists of *gag*, *pro*, *pol*, and *env* genes flanked by long terminal repeats (LTRs). Particularly, the *env* gene encodes for the envelope proteins that initiate the infection process by binding to the host cellular receptor(s), causing membrane fusion. For this reason, a major element in understanding ERVs’ evolutionary trajectory is the characterization of *env* changes over time. Most of the studies dedicated to ERVs’ *env* have been aimed at finding an “actual” physiological or pathological function, while few of them have focused on how these genes were once acquired and modified within the host. Once acquired into the organism, genome ERVs undergo common cellular events, including recombination. Indeed, genome recombination plays a role in ERV evolutionary dynamics. Retroviral recombination events that might have been involved in *env* divergence include the acquisition of *env* genes from distantly related retroviruses, *env* swapping facilitating multiple cross-species transmission over millions of years, ectopic recombination between the homologous sequences present in different positions in the chromosomes, and template switching during transcriptional events. The occurrence of these recombinational events might have aided in shaping retroviral diversification and evolution until the present day. Hence, this review describes and discusses in detail the reported recombination events involving ERV *env* to provide the basis for further studies in the field.

## 1. Introduction

*Retroviridae* is a widely distributed family of RNA viruses that follows a replication cycle involving the reverse transcription of viral single-stranded RNA (ssRNA) into double-stranded DNA (dsDNA), which is inserted into the genome of host cells, thus enabling the expression of viral genes. Usually, retroviruses infect somatic cells but the possibility of infecting germ line cells provides a means for the colonization and fixation of retroviruses into the host genome. Such integrated retroviruses, a constitutive element of the organism genome, are termed “Endogenous Retroviruses” (ERVs) [1,2]. ERVs’ fate, their loss or persistence in the gene pool, depends on random genetic drifts and natural selection since, once integrated into the genome, ERVs follow the Mendelian inheritance pattern and accumulate in the host and thus provide a “fossil” record for ancestral retroviral diversity and their evolution within hosts [3]. One prominent example is the human ERVs (HERVs), which account for a total of 8% of the human genome. The process of such retroviral endogenization cannot just be considered as an ancient event. The present example of Koala retrovirus endogenization [4] indicates the possibility that the integration of retroviruses into the germ line can take place whenever the spread of retroviruses occurs within a host population. Such an endogenization process is not just limited to infection by one particular class of retroviruses and hence, the endogenization of different viral lineages led to the emergence of different ERV types in all vertebrate genomes accordingly [5]. The classification and nomenclature of the individual ERV groups in different vertebrates might not be fully resolved, but the similarities and differences of ERVs to exogenous retroviruses help in categorizing them into seven genera based on their phylogenetic relatedness, i.e., Alpharetroviruses, Betaretroviruses (Class II), Gammaretroviruses (Class I), Deltaretroviruses, Epsilonretroviruses, and Spumaretroviruses [6,7]. The Spumaretroviruses (Class III) are further categorized into five genera, Á., Bovispumaviruses, Equispumaviruses, Felisspumaviruses, Prosimiispumaviruses, and Simiispumaviruses [8]. Apart from the nomenclature, such classifications have also been of great help in understanding the structure of ERVs [9]. In the current review, we first describe the structure and possible role of ERVs’ *env* gene followed by exploring the role of recombination in its diversification.

## 2. Structural Features of Retroviruses

**Figure 1:**
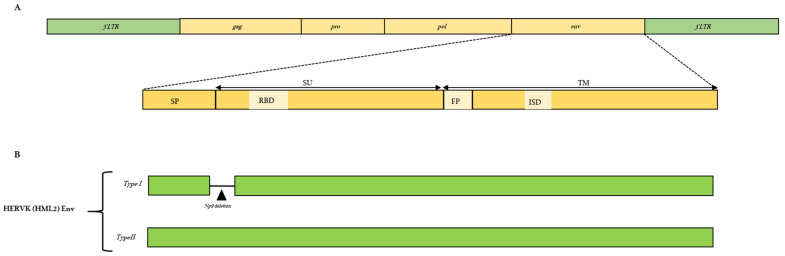
(**A**) General structural features of endogenous retroviruses with a focus on the envelope protein and their main functional domains, i.e., signal peptide (SP), surface unit (SU), receptor-binding domain (RBD), fusion peptide (FP), immunosuppressive domain (ISD), and transmembrane unit (TM). (**B**) Structure of HML2 Env types, i.e., type I (Np9) having 292 bp deletions indicated with a black triangle and type II encoding the Rec protein.

Likewise, for exogenous retroviruses, a complete proviral ERV structure possesses the *gag*, *pro*, *pol*, and *env* genes flanked by long terminal repeats (LTRs) at both ends (Figure 1A). The flanking LTRs are identical and are required to regulate transcription, while the internal regions encode viral enzymes and structural proteins. The *gag* gene encodes capsid (CA), matrix (MA), and nucleocapsid (NC) domains; the *pro* gene encodes for viral protease (PR); the *pol* gene encodes for reverse transcriptase (RT), ribonuclease H (RH), and integrase (IN) enzymes; and finally, the *env* gene includes surface unit (SU) and transmembrane unit (TM) domains (Figure 1A). Differently from the exogenous retroviruses, going through genome mutations and selection pressures, ERVs can be present as a still complete structure or, most frequently, as fragmented portions of proviruses, and they can even be present as solo LTR regions, which means that all the internal coding regions of an ERV have been deleted by recombination events [9]. In addition, even though the complete *gag-pro-pol-env* internal coding regions could be present, they are susceptible to mutations such as substitutions, insertions, and deletions, which might cause sequence modifications and eventually loss of function. Of note, the accumulation of gene mutations also relates to the evolutionary timespan since the beginning of ERVs’ insertion in the host germline. Among the four protein-coding genes of ERVs, *gag* and *pol* are considered to be the most conservative, while the *env* gene, on the other hand, is more prone to mutations and, hence, is extremely divergent [5,10]. In order to study ERVs in detail, phylogenetic analyses are performed on the *pol* gene and the TM region of the *env* gene, further comparing the *pol* and the *env* phylogenetic trees to understand the evolutionary patterns. For example, a recent study performed by Chen et al. [11] analyzed more than 30,000 ERV copies of gamma-type Env in all of the fish and amphibian genomes and transcription assemblies. Furthermore, they performed phylogenetic comparisons with the neighboring *pol* gene in order to study the diversification of the *env* gene and to detect any possible recombination event in the *env* gene [11]. Indeed, *env* divergence studies can provide insights into the emergence of retroviral evolution, such as how the new retroviral lineage emerged. Various studies have shown some evidence of cross-species transmission as well as recombination events that might have led to the generation of new viral variants of a particular class.

The retroviral infection process is initiated by the Env glycoprotein encoded by the *env* gene that binds to the host cellular receptor(s), leading to membrane fusion. As mentioned before, the *env* gene encodes SU and TM subunits (Figure 1A). The SU-TM heterodimers are assembled at the cellular membrane to form an Env trimer. The SU subunit consists of a receptor-binding domain (RBD), which is responsible for the recognition of the host cellular receptor(s) and is considered more variable, making it less useful for phylogenetic analyses [12]. This is because the SU is exposed to the host immune system and, thus, is under high selective pressure [12]. While, on the other hand, the TM includes an RBD, fusion peptide (FP), heptad repeats (HR1 and HR2), ISD, and CX(6)C motif [11] and is quite conserved.

Some of the domains in the Env glycoprotein that help in such characterizations and phylogenetic analyses are the presence or absence of an immunosuppressive domain (ISD), a covalent disulfide linkage, and a hydrophobic fusion peptide (FP) which is present at the N terminal of the TM subunit. Thus, along with the RT region of the *pol* gene, the conserved domains of the *env* gene have aided in the classification of ERVs. The Env expressed on the infected cell’s surface also competes to occupy the receptor in order to prevent multiple retroviral infections within the same cell, and such a phenomenon is known as superinfection interference [13,14].

### Co-Option of ERVs’ Env

Co-option is the term used for the evolution of ERVs’ viral proteins, formerly used for viral infection and replication which has been subsequently repurposed to benefit a variety of host biological functions. Various molecular processes, cellular mechanisms, and biological pathways have appeared to have repeatedly benefited from such viral co-option events [15]. One recurrent ERV co-option is related to antiviral functions, in which the ERV protein interferes with any step of viral infection by acting as a restriction factor. To date, various ERV proteins have been reported to confer resistance to viral infections such as EV3, EV6, and EV9, which are three endogenous loci of chickens that provide entry-level blockage to ALV (avian leukosis virus) infection by receptor interference [16]. Two additional examples of restriction factors are *Rcmf* (*Rcmf* and *Rcmf2*) [17,18] and the Fv4 gene, also known as *Akvr-1*. The earlier gene confers resistance to polytropic MLV strains and is incapable of expressing infectious viruses, while the latter, i.e., the *Fv4* gene, confers resistance to infection by ecotropic murine leukemia virus (MLV) in laboratory mice [19]. Studies have revealed that the Fv4 gene consists of a defective MLV provirus with an intact *env* gene but lacks the 5′ half of the provirus as well as 5′LTR, and hence, its expression is mediated by the cellular genes close to the proviral insertion [20]. Until recently, no human ERVs were reported to be involved in resistance to infections caused by current exogenous retroviruses. However, recent studies have reported the antiviral effect of the HERV-T and HERV-R *env* gene. A study demonstrated the antiviral activity of *HasHTenv*, which is a fusion-defective HERV-T *env* in the human genome [21]. In order to understand *HasHTenv*’s role in entry restriction, a functional HERV-T Env was reconstructed and used for the identification of the entry receptor. The *HasHTenv* was observed to block the infection by virions consisting of a fully functional HERV-T *env* by receptor interference [21], thus suggesting that this HERV-T *env* might have evolved over time and might have led to the extinction of the retrovirus that infected our ancestors [21]. In addition to HERV-T, a recent study reported the antiviral activity of the HERV-R *env* gene [22]. The overexpression of HERV-R Env showed an inhibitory effect on SARS-CoV-2 replication, and its silencing promoted viral replication. HERV-R Env has been previously reported to stimulate the immune system and trigger inflammatory pathways. It has also been reported to be involved in autoimmunity and to be upregulated in many cancers. The study showed that the HERV-R Env activates the ERK pathway which controls the synthesis and activation of AP1 transcription factors such as c-Fos and c-Jun. One of the reasons for the ERK pathway’s activation is that the HERV-R Env contains the CKS-17-like immunosuppressive motif that is responsible for the activation of this particular pathway [23,24]. Even though the exact mechanism of SARS-CoV-2 inhibition by the HERV-R Env remains unclear, there is a clear indication of an inverse correlation between HERV-R Env protein levels and viral load. Hence, these recent findings highlight a possible evolutionary benefit of ERVs in the human genome.

One of the most studied co-opted ERV Envs are the “syncytins” proteins that are expressed in the human placenta and are known to play a crucial role in human development and thus contribute to placental syncytial structures. The syncytins are of major interest because of their domestication by hosts in different mammal lineages that co-opted different retroviral env genes for placentation [25]. The two well-characterized syncytins are syncytin-1 and syncytin-2, which are encoded by HERV-W (ERVWE1) and HERVFRD-1 (ERVFRD-1), respectively [26]. ERVWE1 was first acquired in primates approximately 25 million years ago, and while it has coding-defective *gag* and *pol* genes, it retains the *env* ORF, producing a protein with pregnancy-related functions, i.e., syncytin-1 [27,28]. Syncytin-1 is a 538 amino acid (aa) protein located at the 7q21.2 locus of the human chromosome consisting of a signal peptide (SP), SU, and TM [29,30]. It is highly fusogenic and actively involved in trophoblast cell fusion and differentiation, thus playing an important role in human morphogenesis, which is crucial in placental functions along with its immunomodulatory activity during pregnancy [29]. Interaction with the type-D mammalian retrovirus receptor known as hASCT-1/2 (human sodium-dependent neutral amino acid transporter type 2) aids in the activation of syncytin fusogenic activity [26,30,31]. Ever since the characterization of syncytin-1, its expression in various pathological conditions has been studied to understand its role in diseases such as multiple sclerosis and different types of cancer [31]. Similar to synctin-1 is syncytin-2 Env, encoded by a HERV-FRD provirus located on the 6p24.1 locus of the human chromosome and present in the species of both *Catarrhini* and *Platyrrhini* parvorders, indicating that its acquisition occurred more than 40 million years ago. It also has all the same functional domains as that of syncytin 1, coding for a 538 aa protein. Similar to syncytin-1, syncytin-2 also codes for 538 aa and is expressed as a precursor that is associated with forming homotrimeric complexes and has the same domains as that of syncytin-1.

Syncytin-2 is also required for functional placental syncytia and is expressed in villous cytotrophoblasts [32]. The receptor identified for syncytin-2 is the major facilitator superfamily domain containing 2a (MFSD2a), which is a transporter for an essential omega-3 fatty acid [26,32,33]. The syncytins are an example of convergent evolution, having evolved independently across the mammalian lineages. Even though not related to convergent evolution, some other Env products that are also detected in placental trophoblasts are ERVV-1, ERVV-2, ERVH48-1, ERVMER34-1, and ERV3-1 [34]. Of note, syncytins along with the other mentioned ERVs belong to the class I gamma-type Envs. In order to develop animal models to investigate the role of syncytins in placental development, syncytin-encoding genes have also been searched for in mouse genomes. This led to the identification of two retroviral envelope proteins, i.e., syncytin-A and syncytin-B [35]. Even though the syncytins identified in mouse genomes are different from that of the human genome, they still share the same characteristics, i.e., being specifically expressed in the placenta, having fusogenic properties, and being conserved since their integration into the ancestor mouse genome. In mice, the fetal capillaries are separated from the maternal blood lacunae by two different layers of syncytiotrophoblasts (ST-I and ST-II) [36]. Although distinct from the human placental structure, the ST-I and ST-II layers of the mouse placenta are still proposed as being functionally analogous to the single syncytiotrophoblast layer of the human placenta. Syncytin-A was found to be expressed in ST-I while syncytin-B was detected in the ST-II layer. Both layers coordinate to preserve the structural and functional integrity of the maternal–fetal interface [25]. Even though primate and muroid syncytins share similar characteristics, they are not orthologous and are the result of independent gene capture events in each lineage. The fifth syncytin identified is *Syincytin-Ory1* [37]. *Syncytin-Ory1* was identified in the Leporidae family, i.e., rabbits and hares. This syncytin gene encodes a placenta-specific Env protein with fusogenic activity that is conserved for over 12 mya. Syncytin-Ory1 has the same receptor as that of the human syncytin-1, i.e., ASCT-2 [37]. Its expression was detected in the placental junction zone, where the placental syncytia come into contact with the maternal decidua, suggesting putative involvement in syncytiotrophoblast function. A more recent functional syncytin gene reported was the sixth gene, i.e., *syncytin-Car1*, which was detected in 26 carnivore species [38]. Carnivores belong to the superorder Laurasiatheria and diverged from Euarchontoglires more than 80 mya. Hence, *syncytin-Car1* is the oldest syncytin gene reported so far [38]. The seventh syncytin gene was the most recently discovered in the suborder Ruminantia, in those species that lack the syncytium but display synepitheliochorial placentation [39]. The cell fusion process is very limited, in which only trinucleated cells are being formed, with evidence of heterologous fusion between cells of fetal and maternal origin, a feature that is not found in any other eutherian mammals. This was identified in cows and termed as *syncytin-Rum1*. This syncytin was also detected in sheep genomes as well as 14 other higher ruminant species, indicating that this gene has been conserved for more than 30 mya [39]. Overall, these studies suggest that the retroviral *env* genes have been co-opted independently through the course of evolution.

Apart from the gamma-type Envs, one of the most studied Env proteins among the class II or beta-type ERVs is the HERV-K group HML2, which has been active and infectious for about 30 million years and makes up to 1% of all of the classified ERVs [40]. The envelope of HML2 has been divided into two subtypes, i.e., type I and type II, based on the presence or absence of 292 bp deletions in the *env* gene, respectively (Figure 1B). Accordingly, the two types provide alternative splicing variants of *env*: type I encodes for the Np9 protein in the SU region that predominantly shows nuclear localization and is suggested to act as an oncoprotein that interferes with the PLZF (promyelocytic leukemia zinc-finger protein) repression of c-myc [41]. Type II encodes a Rec protein which is similar to the Rev protein of human immunodeficiency virus (HIV) and the Rex protein of human T cell leukemia virus (HTLV) [9,42]. It has been shown that an HML2 Env is expressed in villous and extravillous cytotrophoblasts during the whole gestation period but is found in neither placental syncytiotrophoblasts nor associated with any specific HML2 provirus at the genomic level [43]. Since HML2 proviruses are evolutionarily the youngest HERVs, their residual activity has gained a significant amount of attention, and various studies have focused on expressing their Envs to better understand their potential role in physiological and pathological conditions. Apart from HERVW, HERV-FRD, and HML2, the Env of other ERV groups even though known is not very well understood, and hence, its characterization might help in unveiling its possible functional roles [26].

## 3. Retroviral Envelope Diversification from the Recombination Point of View

As discussed above, ERV Env studies are mostly dedicated to finding its potential role in physiological and pathological conditions. However, focusing on how various factors might have an effect on the divergence of the *env* gene may lead to a better understanding of ERVs’ evolutionary dynamics. The two main sources of genetic diversity within viruses are mutations and recombination. While mutations result in a change in nucleotides, recombination allows for the movement of variants across the genome, thus producing new haplotypes. Recombination does not create new mutations but new combinations of the existing ones. In general, when viral genomes are in the same host cell and two of them exchange the genetic portions, this is referred to as recombination [44]. It is a ubiquitous process generating diversity among viruses and has a major impact on viral evolution. To the present day, different types of viral recombinants have been identified based on their structure and crossover sites such as homologous recombination [45], non-homologous recombination [45], shuffling, or reassortment [44]. Some of the consequences of recombination are the emergence of new viruses and viral strains, leading to the expansion of viral host range, increases in virulence and pathogenesis, resistance to antiviral strategies, etc. [46,47]. Thus, having insights into *env* recombination events over time can shed light on ERVs’ modification and evolution within the vertebrate genomes. Some of the retroviral recombination events that might have taken place include the acquisition of *env* genes from distantly related retroviruses, *env* swapping facilitating multiple cross-species transmission over millions of years, ectopic recombination between the homologous sequences present in different positions in the chromosomes, and template switching during transcriptional events. Furthermore, in this review, we will explore some of the possible recombination events in detail and understand their impact on shaping retroviral diversification.

### 3.1. ERV–XRV Recombination by Template Switching

**Figure 2:**
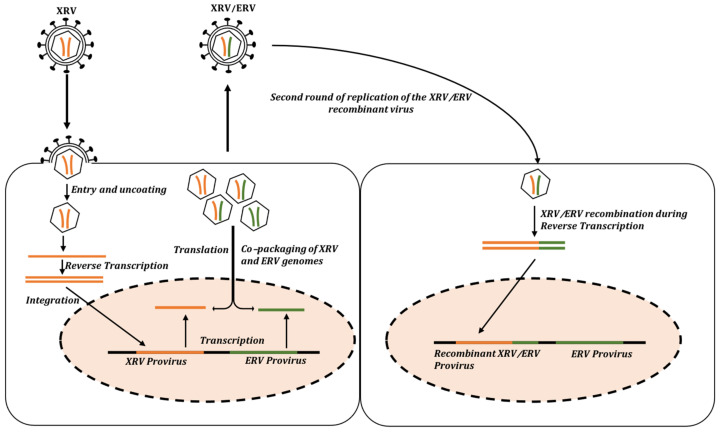
Schematic Representation of an XRV–ERV recombination event. The interaction between the XRV and the ERV leads to the emergence of a recombinant virus. During the first round of infection, the XRV–ERV viral RNAs are co-packaged into the same virion and are further integrated into the host genome during the second round of viral replication.

ERVs are fixed in host populations and are markers of ancient infections by exogenous retroviruses (XRVs). Most of the ERVs are not direct counterparts of the currently circulating, modern XRVs but are phylogenetically related to the existing ones. The interactions between ERVs and XRVs are a phenomenon that has shaped the biology and evolution of retroviruses and provides an opportunity to study host evolution and witness the probable past, present, and future of ERVs’ endogenization (Figure 2) [48]. Such ERV–XRV interactions often hint towards the emergence of recombinant variants that usually occur during the retroviral replication cycle, when the related viral RNAs are co-packaged in virions (Figure 2) [48].

The recombinant variants generated through such events may modify the natural cellular tropism, leading to alterations in receptor binding and immunological self-tolerance. These kinds of ERV–XRV exchange or vice versa can impact the pathogenicity of retroviral infections.

A prominent example of ERV–XRV interaction is *env* recombination in mice, i.e., the recombination between the exogenous MuLV and the members of endogenous retroviruses of mice. The mice endogenous retroviruses are an extensively studied group of retroviruses. During the lifetime of the host mice, these retroviruses—like HERVs—are expressed in a controlled fashion which means that they are incapable of producing infectious viruses, regardless of the presence of the open reading frame coding regions [49]. During the process of mice being infected by exogenous ecotropic murine leukemia viruses (E-MuLVs), it was observed that they frequently recombine with members of a group of endogenous proviruses, generating the recombinant polytropic MuLVs (Table 1). Such an event leads to the substitution of the E-MuLVs *env* gene RBD region with a similar *env* gene region of endogenous P-MuLVs [50]. These recombinant viruses thus utilize a different cell surface receptor for their entry, altering the host range and increasing their pathogenic potential (Table 1) [50,51]. One of the possible mechanisms involved in the formation of recombinant P-MuLVs is the co-packaging of a genomic RNA heterodimer consisting of one transcript of each E-MuLV and P-MuLV in the virion [52]. When this virion infects the cell, recombination between the two RNAs can occur by a copy choice mechanism that involves template switching during the reverse transcription process of the heterodimer. The selectivity of the viral progeny towards this recombinant RNA genome depends on various factors such as changes in the transcription levels of the proviruses, the ability of co-packaging heterodimeric RNAs, or the ability to efficiently transcribe the heterodimers during infection [49,53].

Another well-characterized ERV–XRV interaction is observed in feline leukemia virus (FeLV), belonging to the gammaretrovirus class that infects domestic and wild felid species with various disease outcomes. Multiple felid species are susceptible to FeLV infection but, to the best of our knowledge, only cats of the *Felis* genus have been reported to have endogenous FeLV (enFeLV) in their genome [54]. FeLV is a genetically complex virus including various subgroups such as FeLV-A, -B, -C, -D, -E, and -T. The genetic variability of these subgroups is due to error-prone reverse transcription and the recombination between the exogenous and the endogenous form of the virus [55]. The FeLV-A subgroup can be found exclusively as an exogenous virus and is hence the most relevant epidemiologically [56] while the other subgroups are the result of mutations, deletions, and recombinations. Accordingly, the recombination between the *env* gene of enFeLV and exogenous FeLV-A gives rise to FeLV-B (Table 1) [56,57]. FeLV-B incorporates the enFeLV Env RBD which leads to a change from THTR-1 (FeLV-A) to a Pit1/2 (FeLV-B) recognition receptor [58]. Of note, not all enFeLVs encode for an intact *env* gene, and hence, during recombination, a defective or partially defective enFeLV can exchange its *env* gene, giving rise to an FELV-B that can mediate resistance to FeLV by blocking the receptor and thus affecting the viral infection [57,59]. Indeed, such an endo-exogenous virus interaction of FeLV represents a useful system to examine the genetic interactions in normal as well as diseased conditions in natural and experimental settings.

Similar events of ERV–XRV interactions have been proposed for mouse mammary tumor virus (MMTV) betaretroviruses (Table 1), which are transmitted from infected mice to newborns by milk-causing mammary carcinomas in susceptible animals. Mammary tumorigenesis takes place after the integration of proviral MMTV near a proto-oncogene. MMTV insertion is not sequence specific and, hence, the more the virus spreads, the more it will have a chance to integrate near the proto-oncogene [79]. The generation of tumorigenic variants of MMTV is often caused by recombination between exogenous and endogenous MMTVs. This recombination often occurs by strand switching during cDNA synthesis. When the XRV MMTV infects the cells in which the endogenous form is highly expressed, a novel recombinant XRV MMTV can be generated, with it possessing the ability to infect different strains of mice. Such an event was observed by Golokinva T. et al. in 1997 [80], when they reported a new recombinant virus emergence. They found two new exogenous MMTVs of unknown origin in BALB/Ct mouse strains (BALB2 and BALB14) encoding Vβ2- and Vβ14-specific Sags, respectively. The MMTV and its endogenous form Mtv both encode a type-2 transmembrane protein termed Sag that is the viral accessory protein encoded by the 3′LTR and is important for the spread of MMTV from infected gut-associated lymphoid tissue to the mammary glands [81]. The study revealed that an LA virus was generated due to recombination between BALB14 and ***Mtv-****7*, an endogenous form of MMTV. This new LA virus had the ***Mtv-7*** Sag and the mammary gland transcription element from BALB14. While ***Mtv-7*** is expressed only in lymphoid tissue, the LA virus was highly expressed in the mammary glands and had greater T-cell stimulating activity than that of BALB2 and BALB14. Hence, this recombination between BALB14 and *Mtv-7* resulted in better virus transmission and increased virus titer [80].

Overall, a number of genetic recombination events have been reported among the homologous sequences of exogenous and endogenous retroviruses. These recombination events require the co-packaging of both the exogenous and endogenous viruses into a virion as well as a second round of infection and reverse transcription in order to generate the recombinant forms (Figure 2).

### 3.2. Gain and Loss of ERVs’ env Due to Recombination

**Figure 3:**
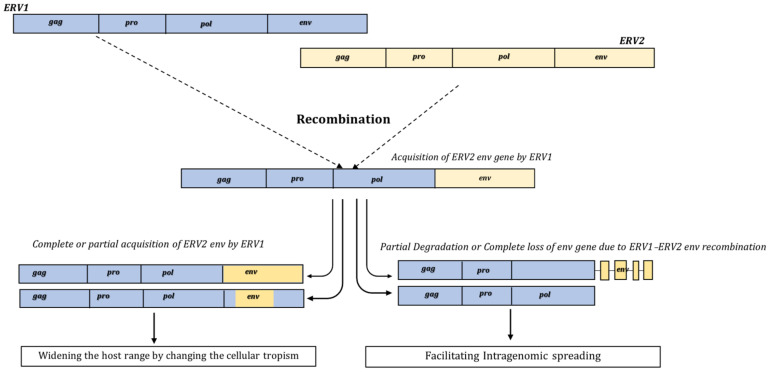
Representation of gain and loss of the Env gene due to recombination. The env acquisition or env degradation is explained by the recombination between ERV1 and ERV2. On one hand, complete or partial acquisition of *env* can lead to changes in the cellular tropism by the change in host receptors, while env degradation or complete *env* can increase the intragenomic spreading of ERVs in the host genome.

Likewise, with the emergence of recombinant variants due to ERV–XRV interactions, *env* exchange among ERVs that belong to different groups, supergroups, or classes is a widespread phenomenon [48]. The two possible recombination events that cause *env* modifications are either the gain or loss of the *env* gene (Figure 3). While on the one hand, the acquisition of the new envelope provides access to the new host population, changing the cellular tropism, on the other hand, the loss of the envelope affects the ability of the extracellular new viral infection and enhances intragenomic spread, thus acquiring a significant amount of the host’s genome [82]. The cases of both *env* acquisition and loss have been well documented in the literature.

#### 3.2.1. Env Acquisition

The patterns of *env* acquisition are observed when a retrovirus acquires a heterologous *env* through the events of recombination. Such acquisition can arise when two different RNA sequences are encapsulated within the same virion [83], as described above for template-switching-mediated recombination (Figure 2). When these recombinations occur, they can result in the emergence of novel retroviruses that can facilitate inter- or intra-species transmission.

A well-characterized case of *env* exchange with Baboon endogenous retrovirus (BaEV) was reported by Chiu et al., 1983 [60] in squirrel monkey retrovirus (SMRV). The SMRV is a New World primates’ gammaretrovirus and is present in the endogenous form with 10–15 DNA copies in the squirrel monkey genome. While analyzing the major *pol* gene progenitors among the type-A, -B, and -C oncoviruses that are the causative agents of naturally occurring tumors in the vertebrate species and are usually transmitted within the germ line of the host, some unusual homology patterns were observed in the *env* genes. These patterns shared similarities with the chimeric type BaEV from Old World primates [84]. Such similarity indicates that it might have diverged from a common ancestor, thus demonstrating the genetic interactions between these viruses’ progenitors and contributing to the evolution of oncoviruses [84]. Since the study initially focused on the *pol* progenitors and eventually identified the env recombination event, the following study was performed by the same group in 2003, focusing on the *env* gene. The study indicated that SMRV and BaEV shared an *env* highly conserved region, suggesting that they became evolutionarily linked to each other due to the recombination of the p15E coding portion [85]. The p15E is a viral envelope protein present in the TM region and has been tested for its potential for immunizing the host species and is considered as a candidate for vaccine development. As described above, this recombination event has been observed not only in the squirrel monkey genome belonging to the *Platyrrhini* parvorder but has also been found in humans and other primates that belongs to the *Catarrhini* parvorder, indicating that these recombination events were established in primates before their split, i.e., around 50–60 mya [84,85].

A similar event was observed by Van Der Kuyl et al. in 1997 [61] while analyzing the simian genomic organization and distribution of endogenous gammaretroviruses. The ERV identified in the study was named simian endogenous retrovirus (SERV), belonging to class I gammaretroviruses. The newly identified ERV highlighted some interesting features of both endogenous and exogenous retroviruses since it showed the high structural similarities of *gag*, *pro,* and *pol* genes with type-D retroviruses, but major differences were observed in the *env* gene. In fact, the gene encoding gp70 protein did not show any similarity with the type-D retrovirus but it was instead homologous to Baboon endogenous retrovirus (BaEV, class I) (Table 1) [61]. Studies have indicated that BaEV proviral sequences are found in African monkeys and were integrated into the host germline around 24–400 thousand years ago, which is quite young with respect to endogenous retroviral evolution. It was speculated that BaEV might be a recombinant virus itself [62] given that all the other type-D retroviruses were found in all monkeys but the BaEV was circulating in only a subset of African monkeys [63]. Later, a study proposed that BaEV is actually a chimeric gammaretrovirus that emerged from a recombination event followed by co-infection involving *Papio cynocephalus* endogenous virus (PcEV), which might be one of the ancestors contributing the *gag–pol* regions to the chimeric BaEV (Table 1) [63,86]. These findings suggest that due to such a recombination event in the *env* gene, a new primate retrovirus evolved in the recent past. In line with the emergence of chimeric BaEV followed by *env* swapping with SERV, another viral variant that emerged during the same time frame is a domestic cat ERV designated as RD-114 retrovirus [54]. Similar to BaEV, RD-114 is also a chimeric virus, with it having the *gag–pol* of ERV of domestic cats (ERV-DC) and the *env* region of BaEV (Table 1) [87]. The acquisition of SERV *env* by PcEV, producing BaEV, and secondly the capturing of BaEV env by ERV-DC aided in the generation of RD-114 [54,86,87].

A very recent example of Env acquisition is the recombination between ERVs of class III spumaretrovirus and class I gammaretrovirus which leads to the emergence of novel XtERV-S. An intact ERV, i.e, Xenopus tropicalis (XtERV-S), was identified in Anuran amphibians by Yedavalli.V et al. in 2021 [64]. This newly reported ERV showed an unusual domain relationship to the known retroviruses, with it having close sequence homology of ***gag–pol*** genes with the ancient class III ERVs while the ***env*** gene showed major differences. The SU of the ***env*** gene did not show a close relationship with any of the known retroviruses and the TM region was observed to be homologous to gammaretroviruses based on its organization and functional motifs [64]. The CWIC motif present in the TM region of XtERV-S has the potential to establish a covalent bond with its gamma-like TM ***env***. The gamma-like envs are sub-grouped based on the “stutter” in the N-terminal heptad repeat. Hence, this motif present in XtERV-S shares similarities with some alpharetroviruses, the ERVs’ of spiny-rayed fish, and some syncytins [88]. The stutter has assumed a functional role in entry mechanisms that involve endocytosis, thus having important functionality and ancient origins that are supported by its presence in the env gene of filoviruses, arenaviruses, influenza viruses, and coronaviruses [89]. As described above, multiple events of ***env*** exchange recombination have been observed between class I and class II retroviruses in different species, but XtERV-S is an unusual example of an intact and non-defective ERV genome having the class III ERV backbone with class I ***env***. Therefore, the XtERV-S might represent the ancient evolutionary retroviral form with a combination of viral genes that are not present in modern mammalian retroviruses but are still circulating in African frogs [64].

A study performed by Vargiu et al. [9] also reported the occurrence of recombination events in HERV, which suggests that recombination is the source of mosaicism of ERVs and frequently occurs either in class I or class II elements. Among class I HERVs, the Harlequin seems to be most prone to recombination, as its structure itself is composed of parts of different HERV sequences, i.e., LTR2-HERVE-MER57I-LTR8-MER4I-HERVI-HERVE-LTR2. Among class II HERVs, HML1, HML2, and HML3 are most frequently involved in recombination. For that of the class III ERVs even though no extensive work has been carried out, the same study suggested that “*env* snatching”, i.e., the loss of *env* as described below, is a very common strategy of intragenomic spreading [9]. As we know, divergence in the *env* gene plays an important role in determining the host range due to the change in the receptor and also through the emergence of a new variant that can infect a different species as in the case of RD114 that can infect not only feline cats but also humans and primates as well as dog species [90], as can clearly be observed from all of the mentioned cases.

#### 3.2.2. Env Degradation

The adaptation of retroviruses in becoming intracellular retrotransposons often leads to either degradation meaning that the *env* gene contains several deletions and eventually does not encode complete Env or loss, i.e., the complete absence of the *env* gene which increases intracellular mobility but diminishes the interhost infection and, hence, leads to the termination of replication (Figure 3). One such case is the presence of *env* less intracisternal type-A particles (IAPs), a group of beta-type ERVs present especially in rodent lineages [65]. Another group is IAPEs (intracisternal type-A particles elements with envelope) that, having an intact *env*, were initially considered IAP progenitors. Later studies showed that this is not the case and that IAPs were independently acquired by mouse genomes [82]. Even though possible recombination mechanisms for their loss of *env* have not been reported yet, they are considered “intragenomic superspreaders” [82]. Superspreaders are the elements that reach a high copy number in the host genome (Table 1, Figure 3).

A similar kind of event was observed in the koala retrovirus (KoRV), the most recent ERV that is currently under the process of endogenization in koalas, with it being integrated into the koala population of Northern Australia while it has not yet been fully integrated into the rest of the southern population [91]. By far, 10 subgroups of KoRV have been characterized, out of which KoRV-A is present in the endogenous form, inducing immune tolerance to KoRV by inhibiting the natural production of antibodies against the exogenous virus [92,93]. KoRV is thought to spread both horizontally by infection as well as vertically in its endogenous form, but it is not present in all members of the host species. For KoRV-A, a variant termed as recKoRV1 was generated due to recombination with an older degraded retroelement designated as PhER, i.e., *Phascolarctos* endogenous retroelement (Table 1) [91]. It has been suggested that the recombination with PhER mediates the degradation of the *env* gene, and hence, the recombinant-derived KoRV might suffer loss of virulence as no part of the recKoRV encodes for an intact virus [66]. Since parts of the defective PhER are incorporated into recKoRVs, these recombinants might exert potential deleterious effects once they are inserted into the host genome and might reduce the intactness of KoRV, thereby decreasing its ability to produce infectious particles. Thus, it can be suggested that disruption of *env* intactness due to recombination might be one of the aspects of the switch of a provirus from horizontal to vertical transmission [66].

Another recently well-documented case of *env* loss is the recombination event between the *env* gene of HML2 and the MER11A LTR of HML8, specifically in macaque species (Table 1). During HML2 characterization in two macaque species, i.e., *Macaca fasicularis* and *Macaca mulatta*, an *env* swapping event was observed in a cluster of 81 HML2 sequences [67]. In this recombination event, the HML2 *env* was almost completely replaced by MER11A LTR of HML8, causing *env* gene disruption [67]. The fact that several copies of this recombinant proviral variant were independently integrated into the macaca genome suggests that the event likely occurred during the process of endogenization, which eventually led to the intragenomic emergence of a separate HML2 *env* type specific to macaques, as no such events were observed in the human HML2 loci [67]. Hence, while *env* swapping is a widespread phenomenon often leading to the emergence of newer variants, recombination events like HML2-HML8 (MER11A) often cause the loss of the *env* gene, resulting in the fixation of fewer *env* retroviruses in the host genome [67,82]. Overall, from the above-mentioned findings, it can be interpreted that both the gain and loss of the *env* gene can be proven beneficial by increasing the fitness of ERVs, and hence, tracing these events can help in exploring evolutionary dynamics and can also have an impact on the emergence of novel variants, thus expanding the host range with the help of cross-species transmission (Figure 3).

As we know, ERVs appear to have a complex evolutionary history and have been fixed in their hosts through replication, reinfection, and, over time, expansion in the genome by intracellular retrotransposition mechanisms. Apart from retrotransposition, ERV elements also undergo unequal crossing over and gene conversion termed as “ectopic recombination” and, hence, are being hypothesized to be the major contributors to host genome plasticity [94]. Even though no specific ectopic recombination events have been reported in the *env* gene, studies have revealed the breakpoint for gene conversion and recombination in HERV-H and HERV-K elements [94]. Therefore, even if the exogenous viral counterparts of ERVs have become extinct, they have managed to significantly expand their copy number throughout evolution by the above-described *env* exchange mechanisms.

### 3.3. Cross-Species Transmission by Env Recombination

**Figure 4:**
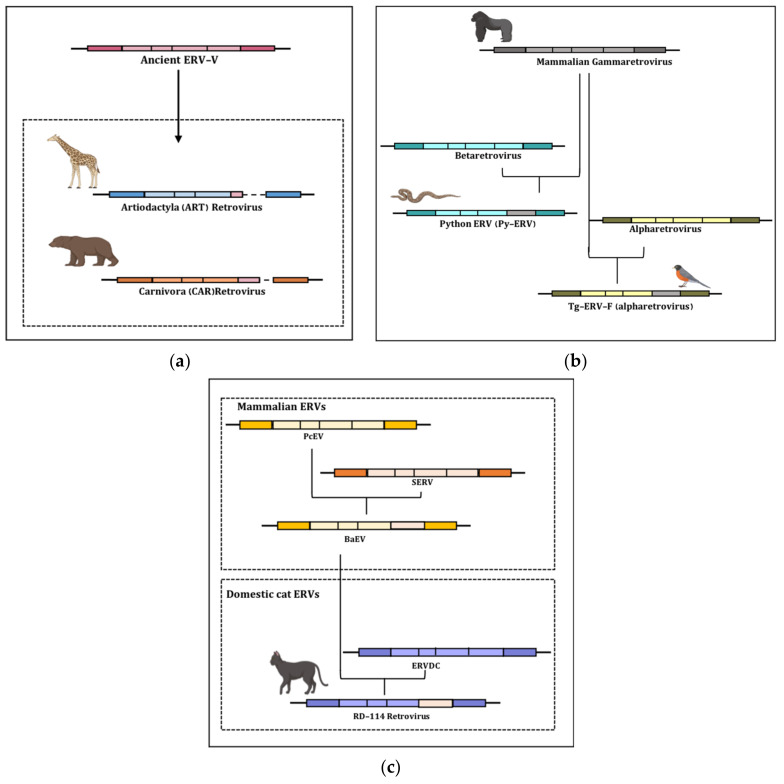
Cartoon depiction of the examples of cross-species transmission of ERVs from one species to another. The figure highlights the general structure of ERVs in different species with the possible recombination event in the env gene. (**a**) ERV-V env swapping between the two-mammalian order Artiodactyl and Carnivora leads to the presence of a partially functional ERV-V env gene in new ART and CAR ERVs that are absent in other mammalian orders. (**b**) The emergence and circulation of alpha–gamma- or beta–gamma-type ERVs in various species such as songbirds and pythons, respectively, as a result of env recombination. (**c**) Series of recombination events in mammalian ERVs and feline ERVs leading to the emergence of a chimeric RD-114 retrovirus.

Interestingly, a third factor that plays an important role in retroviral *env* diversification is cross-species transmission and the recombination events associated with such jumping across vertebrate species, which represents a major source of emerging diseases (Figure 4). A prime example of cross-species transmission in retroviruses is the pandemic caused by HIV-1 group M that followed the transmission of SIV from chimpanzees to humans in the early 20th century [95,96]. Similarly, various other cross-species transmission events have been reported between humans and other primate species. Apart from transmission between humans and primates, other retroviral transmissions have also been observed in the mammalian species, some of which have resulted in endogenization (Figure 4). Hence, studies on ERVs have provided a great opportunity to understand the cross-species transmission that might have occurred during the course of evolution. In the above sections, we reported some recombination events leading to *env* swapping between different ERV classes within the same vertebrate lineage. Surprisingly, such events can also involve the swapping of retroviral elements in distantly related hosts. One of the major ways of understanding the cross-species transmission of viruses is broad-scale genomics and phylogenomic analysis. A recent phylogenomic study performed to understand retrovirus–host evolution also suggested some events of cross-species transmission. A methodology was developed to identify the phylogenetic signals from large ERV datasets across 60 vertebrate species [97]. The findings indicated a history of frequent horizontal interorder transmission of gammaretroviruses and other associated class I retroviral sequences from rodent reservoirs. The phylogenetic patterns might represent a particular mode of evolution for gammaretroviruses, as these retroviral sequences have been reported to occur adjacently in diverse mammalian species, and thus, the gammaretroviruses have the capacity to switch across diverse mammalian hosts [97]. In general, such broad-scale analysis provides a great opportunity to study cross-species transmission events in a wide range of mammals. As an example, focusing on ***env*** recombination, a few cross-species transmission events highlighting ***env*** gene recombination are discussed below.

A recent study reports the cross-species transmission of an ancient endogenous retrovirus in two mammalian orders, i.e., Artiodactyla (ART) and Carnivora (CAR) [98]. The study identified two non-orthologous ERV *env* genes named ARTenvV and CARenvV that were absent in other mammalian orders. These two *env* genes lack the complete TM region while showing positive selection of the SU region (Figure 4a). Thus, the findings suggest that ARTenvV and CARenvV might have evolved independently from a common gamma-type exogenous retroviral ancestor that was cross-transmitted among the two different mammalian orders at least 64 mya [98].

An interesting example is the detection of the mammalian gammaretrovirus *env* gene in the Tg-ERV-F provirus, an ERV of songbirds belonging to alpharetroviruses (Figure 4b). The mammalian origin of this TgERV-F *env* is suggested by the lack of similarity between its sequence and that of the previously characterized gammaretroviruses in the avian species such as chicken retrovirus 1 (ChiRV1) and reticuloendotheliosis viruses (REVs), indicating the possibility of separate transmission events of TgERV-F (Table 1) [68]. Indeed, TgERV-F is the first characterized alpharetrovirus having acquired a gamma-type *env* and hence, it has been able to circulate among various avian species over the last 4 million years.

A study was performed by Chen and colleagues in 2019 [99] to investigate mammal–avian cross-species transmission and identified the presence of mammalian *env* genes in at least 15 avian species, which were divided into two groups: group 1 (recombination between alpha-type *pol* and gamma-type *env*) and group 2 (recombination between gamma-type *pol* and *env* genes). Thus, the study uncovered long-term bird–mammal retroviral interactions. Another case of gamma-type *env* acquisition was observed in python ERVs (PyERV), whose *gag*–*pol* regions align more closely to betaretroviruses while their *env* is more related to murine gammaretroviruses (Figure 4b, Table 1) [69].

A most striking case of cross-species transmission is the one performed by the RD-114 and D-type retrovirus (RDR) interference group, i.e., the largest group of retroviruses using the same receptor on human cells, which includes 10 members infecting a wide range of mammals and avian species [100]. RD-114 is itself a chimeric virus, having the *env* gene of BaEV, and it is supposed to be generated by recombination events (Figure 4c). The group members might have different *gag* and *pol* regions, but they share a homologous *env* gene, thus sharing a common cell surface receptor, i.e., ASCT2, which is also the receptor for HERV-W syncytin-1. The fact that the same receptor is shared between syncytin-1 and the RDR interference group indicates that the same Env glycoprotein mediated the endogenization process on multiple occasions in vertebrate lineages [101]. Such an event has recently been studied in a nine-banded armadillo, where an uncharacterized ERV group was reported in the xenarthran ancient lineage of mammals, suggesting that it was acquired approximately 12 mya, since it has a recombinant *env* gene that also recognizes the ASCT2 receptor. Based on this evidence, the ASCT2 transporter can be proven as a successful receptor in ERV endogenization, hence favoring the emergence of numerous virus variants in a wide range of species by recombination [102]. Thus, it can be hypothesized that the RDR interference group *env* might have been swapped multiple times, facilitating numerous cross-species transmission events throughout vertebrate evolution.

Another remarkable example of the emergence of novel lineages as a result of cross-species transmission has recently been reported in polar bears (*Ursus maritimus*). This newly identified ERV is designated as UrsusERV and shows sequence similarities with the Porcine Endogenous Retrovirus (PERV) that is present in all porcines (Table 1) [70]. The UrsusERVs are found specifically in the Ursinae bear species (hence the given name) and the study suggests that they are part of a phylogenetic clade together with pig, gibbon, and koala retroviruses [71]. Due to its relatively young age, it has been hypothesized that the exogenous form of this ERV has been repeatedly circulated among the host population for over two million years from an unknown reservoir, as similarly described for KoRV endogenization [71], and that it might potentially still be in circulation. Even though the study indicates that the UrsusERV is a case of cross-species transmission from an unknown reservoir, a noticeable fact is its similarity with PERV, which itself is a result of the cross-species transmission of precursor retroviruses from different species. PERV further evolved in the pig genome and has gained a lot of attention over the years especially in the field of xenotransplantation [72]. From the above-mentioned examples, one can hypothesize that since the gamma-type *env* is widely distributed in vertebrate species, its acquisition might afford a virus to a new host environment. If the newly emerging recombinant virus is able to further circulate among related species making it adaptable to the new environment, this might have an impact on the evolutionary trajectory of ERVs.

### 3.4. Xenotropic Recombination

**Figure 5:**
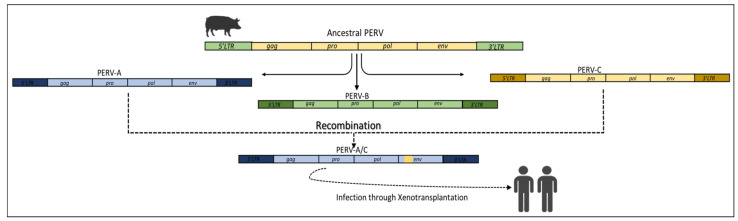
Depiction of xenotropic recombination. The recombination of PERV env which generates the PERV-A/C recombinant. The PERV recombination is not related to the evolutionary process but can infect human cells during the xenotransplantation process.

Xenotransplantation is a medical procedure that might help in reducing the shortage of human organs for transplantation and could serve as a temporary solution. For such procedures, porcine cells, tissues, and organs are often used, but this might increase the risk of zoonotic disease transmission. A way to avoid such transmission is termed designated pathogen-free (DPF) breeding of animals [70,72,73], which does not apply, however, to endogenous forms. Of special importance is PERV, as it is present in the pigs’ genome and hence difficult to eliminate by DPF breeding itself. PERVs belong to gammaretroviruses and are closely related to KoRV, FELV, and MuLV [74]. Depending on the pigs’ breed, three subtypes of PERVs are known, i.e., PERV-A, PERV-B, and PERV-C. Of these, PERV-A and -B are polytropic viruses integrated into almost all pigs’ genomes and also have the capacity to infect different species including humans while the -C subtype is an ecotropic virus that can only infect pigs but is not ubiquitous [75]. The RBD of PERVs contains the two variable regions (VR) VRA and VRB, located between RBD aa 96–126, and a downstream proline rich region (PRR), located between aa 254–298, that is responsible for cellular binding [75]. In PERV-C, the last SU 100 residues are important for binding and infection, and among these, only 9 residues differ from that of PERV-A, suggesting a recombination event between the two (Table 1, Figure 5). Therefore, the presence of these regions determines the PERV tropism. Apart from the transmission of PERVs in human cells during xenotransplantation, the presence of PERVA/C recombinants has also been detected in the HEK 293 cell line. In this recombinant form, the LTR, *gag,* and *pol* genes were of PERV-C, while the *env* gene was derived from the polytropic PERV-A; hence, this recombination allowed the virus to integrate into cells of different organs but not infect the germ line of animals. These recombinants are usually found in minipigs of different origins. The minipigs are the smallest domestic pigs developed for biomedical research purposes [76,77]. A similar study was performed by Klymiuk et al., reporting various PERV recombinant patterns in the *env* gene. In the study, the PERV sequences were designated as PERVγ1 and included PERV-A, -B, and -C subtypes [78]. The study identified 15 distinct recombination patterns in PERVγ1 *env* sequences. Higher recombination patterns were observed in SU and TM, but only a few were identified in RBD, likely due to increased sequence polymorphism in this portion among subfamilies [78]. Several studies have been performed to identify the recombination events among the PERV subtypes in order to have an understanding of how to reduce the risk of transmission to human cells. Therefore, for safer xenotransplantation, it is highly recommended to use PERV-C-free donor pigs as these pigs will be unable to provide it to generate the PERV-A/C recombinants.

## 4. Concluding Remarks

In conclusion, the retroviral envelope has gone through several modifications during the ERVs’ endogenization process, of which one factor was the multiple recombination events that have hence influenced ERVs’ evolution. The recombination in the *env* gene not only leads to the emergence of novel retroviral variants but it also aids in widening viruses’ host range and eventually the co-evolution of these retroviral elements within vertebrate genomes. Overall, it can be inferred that the *env* recombination events highlighted in this review have been a driving force for the genetic diversification of ERVs over the course of evolution.

## Figures and Tables

**Table 1:** Highlights of Env recombination events in endogenous retroviruses.

XRV	Host	Envelope Recombination			References
		Type	Event	Effect	
**MuLV**	Mice	Template switching	XRV E-MuLV—ERV P-MuLV	Alteration in cellular receptor	[49,50,51,52]
**FeLV**	Felines	Template switching	XRV FELV-A—enFELV	Alteration in cellular receptor	[54,55,56,57,58,59]
**MMTV**	Mice	Template switching	XRV MMTV—en Mtv-7	Better virus transmission and high viral loads	[26]
**SMRV**	Squirrel monkey	Env acquisition	SMRV-BaEV	Alterations in highly conserved p15E region	[60]
**SERV**	Simians	Env acquisition	SERV—BaEV	Generation of new recombinant variant	[61]
**BaEV**	Baboon	Env acquisition	BaEV—PcEV	Emergence of chimeric type-C/type-D BaEV retrovirus	[62,63]
**RD114**	Felines	Env acquisition	ERV-DC—BaEV	Generation of RD114	[54]
**XtERV-S**	Xenopus tropicalis	Env acquisition	Class III—Class I ERVs	Acquiring the env gene from class I gammaretroviruses	[64]
**IAP**	Mice	Env loss	ND	Intragenomic spreading	[65]
**KoRV**	Koala	Env degradation	KoRV-A—PhER	Intragenomic spreading	[66]
**HML2**	Primates	Env degradation	HML2-HML8(MER11A LTR)	Intragenomic spreading	[67]
**Tg-ERV-F**	Songbirds	Cross-species transmission	TgERV-F—mammal gamma-type env (unidentified)	Circulation of alpha–gamma recombinant variant in avian species	[68]
**PyERV**	Python	Cross-species transmission	PyERV-Murine gammaretrovirus’ Env	Circulation of beta–gamma recombinant variant in pythons	[69]
**Xenanthran ERV**	Nine banded armadillos	Cross-species transmission	ND	Emergence of novel Xenanthran ERV	[70]
**UrsusERV**	Polar bear	Cross-species transmission	ND	Emergence of novel UrsusERV	[70,71]
**PERV**	Porcine	Xenotropic recombination	PERVA-PERVC	Generation of PERVA/C recombinant capable of infecting human cells	[72,73,74,75,76,77,78]

Abbreviations: murine leukemia virus (MuLV), feline leukemia virus (FeLV), mouse mammary tumor virus (MMTV), simian endogenous retrovirus (SERV), baboon endogenous retrovirus (BaEV), squirrel monkey retrovirus (SMRV), intracisternal type-A particles (IAPs), koala retrovirus (KoRV), human MMTV-like (HML2), songbird endogenous retrovirus (Tg-ERV-F), python endogenous retrovirus (PyERV), and porcine endogenous retrovirus (PERV).

## Data Availability

Not applicable.
